# Epigenetic modulation of myeloid cell functions in HIV and SARS-CoV-2 infection

**DOI:** 10.1007/s11033-024-09266-2

**Published:** 2024-02-24

**Authors:** Carolyn Krause, Eva Bergmann, Susanne Viktoria Schmidt

**Affiliations:** 1 Institute of Clinical Chemistry and Clinical Pharmacology, University Hospital Bonn, 53127 Bonn, Germany; 2 Department of Microbiology and Immunology, the Peter Doherty Institute for Infection and Immunity, University of Melbourne, Melbourne, VIC 3000 Australia

**Keywords:** Epigenetics, Myeloid cells, HIV, AIDS, SARS-CoV-2, COVID-19

## Abstract

Myeloid cells play a vital role in innate immune responses as they recognize and phagocytose pathogens like viruses, present antigens, produce cytokines, recruit other immune cells to combat infections, and contribute to the attenuation of immune responses to restore homeostasis. Signal integration by pathogen recognition receptors enables myeloid cells to adapt their functions by a network of transcription factors and chromatin remodelers. This review provides a brief overview of the subtypes of myeloid cells and the main epigenetic regulation mechanisms. Special focus is placed on the epigenomic alterations in viral nucleic acids of HIV and SARS-CoV-2 along with the epigenetic changes in the host’s myeloid cell compartment. These changes are important as they lead to immune suppression and promote the progression of the disease. Finally, we highlight some promising examples of ‘epidrugs’ that modulate the epigenome of immune cells and could be used as therapeutics for viral infections.

## Introduction

### Myeloid cells

Viruses as a category of pathogens pose a significant threat as they are able to hijack the human host’s cellular machinery and disrupt organ functions, leading to various diseases. The host’s initial response to these threats is through the immune system, which uses viral recognition mechanisms to detect these pathogens, followed by a series of response strategies. Important components of this mechanism are the pathogen-associated molecular patterns (PAMPs), the damage-associated molecular patterns (DAMPs), and the pattern recognition receptors (PRRs). PRRs are expressed on immune cells, such as monocytes, macrophages, and dendritic cells (DCs), to identify PAMPs and DAMPs. PRRs recognize PAMPs, such as specific viral components like viral proteins, nucleic acid, etc., but also other microbial pathogens. In addition, DAMPs, which are cellular components or debris from infected and damaged cells, are also recognized by PRRs [1].

The first line of defense against pathogens in humans is the innate immune response, providing the body’s immediate, nonspecific reaction. This involves myeloid cells like monocytes, macrophages, dendritic cells, and neutrophils, as well as natural killer cells. Additionally, the complement system, comprising a group of proteins, plays diverse roles, including opsonization, chemotaxis, and more. These cells possess phagocytic abilities that allow them to engulf and subsequently degrade pathogens, as well as the secretion of various cytokines, chemokines, and effector molecules. In contrast, the adaptive immune system provides a specialized response tailored to specific pathogens upon the initial infection and to pathogens encountered by the body in the past. At the center of the antigen-specific compartment are T lymphocytes, which can eradicate infected cells and refine overarching immune responses, while B lymphocytes specialize in the precise synthesis of antibodies targeting encountered pathogens. It is noteworthy that DCs and macrophages play a central role in the licensing of T cells, primarily through the intricate process of antigen presentation. The absence of this complicated mechanism, which involves both antigen presentation and licensing, precludes the activation of adaptive immunity, which is essential for effective defense against pathogens [1].

Human myeloid cells (including monocytes, macrophages and dendritic cells) originate from progenitor cells in the bone marrow and constitute a diverse group crucial to the immune system. Their functions range from phagocytosis (e.g., macrophages and neutrophils) to antigen presentation (e.g., DCs and macrophages) and specialized responses against parasites (e.g., eosinophils). Monocytes circulate in the bloodstream for a brief period and differentiate predominantly into macrophages or DCs in response to certain environmental stimuli in tissues, thus acting as precursors of both cell types. With their phagocytic capabilities, monocytes and macrophages capture pathogens and cellular debris from dying cells to prevent inflammatory immune reactions and the development of autoimmune responses. In addition, these cells are key factors in antigen presentation to T and B cells, thereby triggering immune responses. Further, activated monocytes secrete cytokines to attract other immune cells and modulate immune responses. Monocytes are divided into several subsets according to their expression of the two surface markers CD14 and CD16: classical (CD14^++^, CD16^−^), intermediate (CD14^+^, CD16^+^), and non-classical (CD14^−^, CD16^+^). Each subset performs distinct roles ranging from immediate defense responses against pathogens to the inhibition of immune responses and tissue repair. Clinically, abnormal frequencies and functions can indicate disease states, such as chronic infections, autoimmune diseases, and specific leukemias [2].

Macrophages can differentiate from monocytes or derive from tissue-resident macrophages, which originate from yolk-sac progenitor cells [3, 4]. They release specific cytokines that can either promote or restrain inflammation. Further, macrophages have historically been classified into two categories according to their functions in diseases and homeostasis: pro-inflammatory (M1, induced by Interferon-γ; IFN-γ) and anti-inflammatory (M2, induced by Interleukin-4; IL-4) [5]. This simplified model was continuously extended for subcategories such as M2a, M2b, M2c and M2d macrophages based on differences in surface marker expression, secreted cytokines and functional features [6]. Transcriptome analysis of human in vitro differentiated macrophages cultured in the presence of diverse immune activators extended the dogma of M1 and M2 macrophages [7, 8]. Recognition of DAMPs, PAMPs and cytokines with subsequent signal integration resulted in signal-specific transcriptional changes enabled by a network of transcription factors (TFs) [8]. The activated TFs combinations were specific for the incoming signal. These observations lead to the understanding that macrophage activation is of a multidimensional nature, rather than a bipolar model of anti- and pro-inflammatory macrophages. In summary, macrophages show high plasticity and can change their cellular programs and functions in response to signals from the microenvironment [9].

Other groups of human myeloid cells also have pivotal functions in the fight against pathogens. Professional antigen presenting DCs serve as the critical bridge between the innate and adaptive branches of the immune system via processing and presenting antigens to T cells with the aim to induce specialized immune responses. This crucial process serves as a signal for the onset of the adaptive immune response, which develops a specialized defense strategy against specific pathogens [10]. Neutrophils, for example, serve as the body’s primary defense against bacterial infections. These immune cells rapidly respond to sites of infection, where these cells phagocytose and neutralize invading bacteria. In addition, neutrophils release their DNA and antimicrobial proteins to form a web-like structure called a neutrophil extracellular trap (NET), the so-called NETosis process. This NET traps and neutralizes pathogens, serving as a defense mechanism. However, dysregulated NETosis is associated with inflammatory and autoimmune diseases [11]. Eosinophils target parasitic infections and play a key role in mediating allergic reactions. Lastly, basophils, circulating in the bloodstream, and mast cells, are tissue-resident, and are crucial in amplifying allergic reactions by releasing histamine and other inflammatory mediators, even though they are less numerous. In essence, these cells together lay the foundation for a robust and dynamic immune response [12].

### Layers of epigenetic regulation

Proteins, essential building blocks of mammalian cells, are encoded by amino acid sequences in DNA, serving as the genetic code and biological blueprint. This process involves the transcription of DNA into messenger RNA (mRNA), which is then translated into the amino acid sequence that forms proteins. Persistent deviations from this genetic code in the form of single nucleotide polymorphisms (SNPs) are heritable and are considered genetic changes. In contrast, epigenetic changes do not directly alter DNA sequences, but influence gene expression by indirectly altering DNA transcription. Influenced by the environment, aging processes, cancerous processes, dietary effects and lastly acute or chronic infections, epigenetic changes are variable and temporary due to their reversibility. To date, several epigenetic mechanisms have been discovered. Main categories of epigenetic modulation of DNA and chromatin structures refer to (i) DNA methylation, (ii) histone modifications, and (iii) non-coding RNA (ncRNA) [13].

**Fig. 1:**
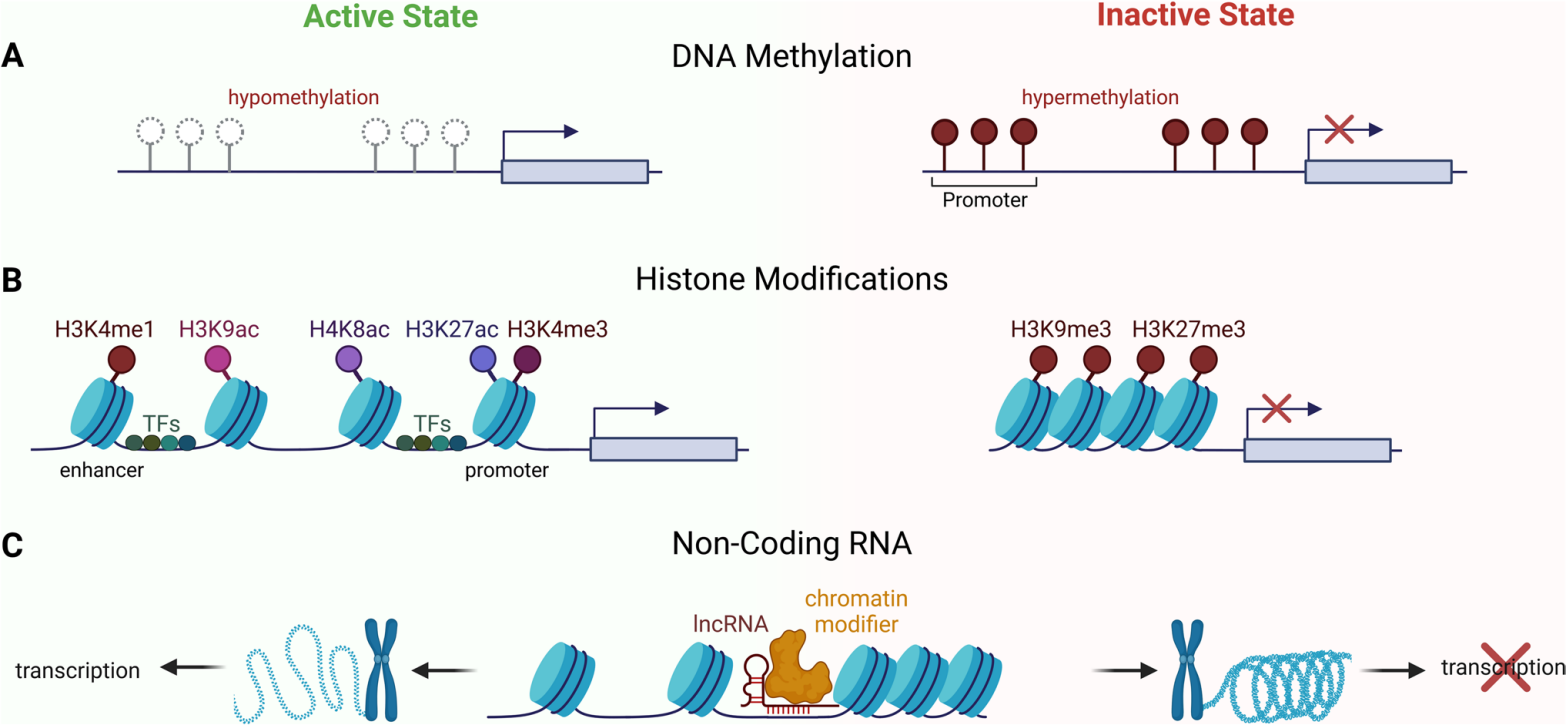
Schematic representation of the main layers of epigenetic regulation. Epigenetic modifications include three main categories: **A** Elevated levels of DNA methylation (hypermethylation) reduce gene expression by inhibiting transcription factor (TF) binding, RNA polymerase activity and promoting a repressive chromatin state in the gene promoter region. Lower levels of DNA methylation (hypomethylation) do not interfere with TF binding and hence are associated with increased gene expression. **B** Chromatin modifications involve alterations in the structure of chromatin, primarily through histone modifications such as acetylation (H3K9ac, H4K8ac, H3K27ac, etc.) and methylation (H3K4me1, H3K4me3, H3K9me3, H3K27me3, etc.), influencing the accessibility for TF complexes and playing a pivotal role in the regulation of gene expression. These modifications can either promote or inhibit transcription by modulating the interaction between DNA and histone proteins. **C** Non-coding RNAs (ncRNAs) play a crucial role in epigenetic regulation by participating in processes like histone modification and chromatin remodeling, acting as key mediators in the modulation of gene expression

DNA methylation is a biochemical procedure wherein DNA methyltransferases (DNMTs) add a methyl group to a specific carbon of a nucleotide base, predominantly at the fifth carbon position of cytosine (C5) within mammalian genomes resulting in 5-methylcytosine (5mC). This modification is frequently observed at 5′-cytosine-phosphate-guanine-3′ (CpG) dinucleotides. Hence, methylation events can be categorized into CpG and non-CpG modifications, with the latter manifesting predominantly in distinct cellular lineages. Regions characterized by a high density of CpG dinucleotides, termed “CpG islands,” play an essential role in the modulation of gene expression. Variations from typical methylation profiles, be it hyper- or hypomethylation, have the potential to perturb gene functionality and are best characterized in oncogenic transformations (Fig. 1A). Promoters marked by 5mC were found to be associated with long-term transcriptional repression and gene silencing [14].

In eukaryotic cells, DNA is organized and compacted around protein complexes known as histones. The compactness of these histone-DNA structures, termed nucleosomes, dictates the accessibility of the transcriptional machinery to the DNA. When nucleosomes are densely aggregated, it is difficult for TFs and other regulatory proteins to interact with DNA, resulting in gene repression. Conversely, a relaxed nucleosome configuration permits increased access to the DNA, facilitating gene activation. The degree of histone compaction can be modulated by specific chemical modifications of the histones, such as the addition or removal of acetyl or methyl groups, which subsequently regulates gene expression by promoting either a repressed or active state [15]. These histone modifications are added to the flexible tails of histones (Fig. 1B). For instance, H3K27me3 describes the tri-methylation of lysine 27 on the histone H3 protein and is a principal modification that typically marks closed chromatin formation, leading to inhibition of transcription. Additionally, H3K9me3, the tri-methylation of lysine 9 on histone H3, is also associated with transcriptional repression and the formation of condensed, inactive chromatin [16]. In contrast, tri-methylation of lysine 4 on histone H3 protein (H3K4me3) marks open chromatin in promoter regions, indicating active regulation of gene expression. Together with the acetylation of lysine 27 at the histone H3 protein (H3K27ac), H3K4me3 also marks accessible promoters in immune cells and active transcription in vitro [17–20]. Another positive histone modification is the methylation of lysine 27 on histone H3 (H3K4me1) correlating with gene activation and open chromatin, particularly in enhancer regions [21]. Moreover, H3K9ac, involving the acetylation of lysine 9 on histone H3, and H4K8ac, entailing the acetylation of lysine 8 on histone H4, also promote a more open chromatin structure and transcriptional activation [20, 22, 23].

As mentioned before, the coding mRNA provides the necessary blueprint for protein synthesis. Within the cellular environment, DNA serves as a template for the synthesis of both coding, ncRNA and long non-coding RNA (lncRNA) with a size of over 200 nucleotides. In contrast to protein-coding transcripts, ncRNAs, such as microRNAs (miRNAs; miR), play a significant role in the regulation of gene expression and are therefore considered an epigenetic regulatory mechanism. They can interact with mRNA molecules, often forming complexes with specific proteins, which leads to the degradation of the mRNA and thus prevents protein synthesis. Additionally, ncRNAs and lncRNAs can facilitate the recruitment of protein entities that modulate histones, thereby influencing the transcriptional state of genes by activating or repressing their expression (Fig. 1C) [24].

There are other epigenetic mechanisms that influence transcription processes that do not belong directly to the three categories mentioned: The secondary structure of DNA can have influences on its methylation state. Guanine-rich regions form stacks of G-quartets that result in DNA folding. These G-quadruplexes (G4s) are recognized by DNMTs or ten eleven translocation enzymes (TETs) and impact the functions of the methylation-regulating enzymes leading to methylation depletion and in consequence gene silencing or at least repression of transcription [25, 26]. So far, the impact of G4s on defense responses against viruses is unclear and remains to be investigated by future studies. Yet, the non-structural protein 3 of Severe Acute Respiratory Syndrome Coronavirus (SARS-CoV) can bind to G4s in 3’-untranslated regions (UTRs) of host mRNAs encoding proteins involved in apoptotic processes and signal transduction [27, 28]. G4s were also found to participate in the recombination of Human Immunodeficiency Virus (HIV) [29], where they are supposed to prevent the degradation of the HIV genome before its integration into the host genome, but moreover might contribute to the unmethylated cytosines in the 5’ long terminal repeat (LTR) region that interfere with transcription observed during latent infected T cells [30, 31]. Further, a recent study demonstrated the importance of G4 structures for the replication and translation of SARS-CoV-2 [32]. Future development of drugs targeting G4 structures may present a promising antiviral therapeutic approach.

In the following chapter, we will give examples of epigenetic mechanisms playing a pivotal role in the host’s defense against viruses with a focus on myeloid cell function.

## Epigenetic influences on myeloid cell function in viral infections

Under inflammatory conditions, human monocytes differentiate in dependency of signals from the microenvironment into DCs or macrophages. The question of whether these two categories of cell types can be distinguished from each other is still debated, as they have similar functions and the identification of the cell type and further subtypes in the past was based on cell surface marker expression quantified by multicolor flow cytometry. Observed differences between human and murine myeloid cells add another layer of complexity to our understanding of myeloid cell types and their functions. Single-cell RNA sequencing (scRNA-seq) helps to precisely define each cell based on its transcriptomic fingerprint and place it in the context of the identity of other myeloid cells. ScRNA-seq in combination with information on open chromatin regions determined by single-cell Assay for Transposase-Accessible Chromatin sequencing (ATAC-seq) is currently improving our understanding of the molecular mechanisms of cell differentiation and activation.

### Mechanisms of myeloid cell differentiation

Differentiation of monocytes by combinations of growth factors and cytokines into DCs and macrophages as in vitro model systems has been extensively studied in the past [33]. Pioneer factors are proteins that bind to target sites of condensed chromatin and open up the chromatin structure [34]. The open chromatin structure enables the recruitment of chromatin remodelers and TFs that prepare the chromatin structure for the induction or repression of gene expression. During the differentiation of monocytes into DCs, the pioneer factor Early Growth Response 2 (EGR2) was found to interact with the 5mC hydroxylase TET2, resulting in DNA demethylation and enabling gene transcription by TF complexes [35], including TFs like IRF4, AHR, STAT6 and PU.1 [36]. Interestingly, TET2 also has an important function in suppressing the expression of pro-inflammatory cytokines such as IL-6, IL-1β and ARG1 in macrophages [37]. Various chromatin remodelers were found to participate in the activation of DCs. For example, the expression of surface markers and co-stimulatory *Cd80* (B7-1) and *Cd86* (B7-2) was found to depend on H3K27 demethylase JMJD3, which renders both gene loci into an active state [38]. The action of JMJD3 could be supported by other chromatin remodelers like the histone deacetylases 1 (HDAC1) and HDAC2. Both were found to influence the expression of *Cd80* and *Cd86, as well as* other direct target genes involved in DC activation, after being recruited to the gene loci by the methyl-CpG-binding protein Mdb2 [39]. The timing of signal integration also seems to be pivotal for the cellular phenotype. For example, the timing of signal integration via IL-4 during monocyte differentiation is decisive for the development of an M2-like or DC-like phenotype [40]. Simultaneous treatment of monocytes by IL-4 and Granulocyte-macrophage colony-stimulating factor (GM-CSF) lead to the activation of the platform protein Nuclear Receptor Corepressor 2 (NCOR2) and the differentiation into a macrophage-like phenotype. Similarly, a recent study described that only the simultaneous signal integration of IFN-α and anti-CD40 can increase the transcription of genes required for the activation of T cells in antiviral immune responses, exemplified in SARS-CoV-2 infection [41]. These two examples demonstrate that our current understanding of differentiation, transcriptional, and epigenetic changes is complex and must be interpreted in a microenvironmental and temporal context.

In addition, Chromatin Immunoprecipitation Sequencing (ChIP-seq) analysis of H3K4me3, H3K27ac and H3K4me1 marks, which can be used to identify active and repressed promoters and enhancers, showed that the chromatin of IFN-γ (M1-like) and IL-4 (M2-like) activated macrophages is accessible [19], enabling the induction of gene expression without prior chromatin remodeling in case of infection. In contrast, tissue-resident macrophages displayed activation of specific TFs enabled by tissue-specific promoters marked by permissive histone modifications H3K4me3 and H3K27ac, and active enhancers marked by H3K4me1 and H3K27ac [19, 42].

### Trained immunity in myeloid cells

In recent years, multiple concepts have been proposed as to how myeloid cells can adapt adequately to the microenvironment, shape their immune response to the invading pathogen and prepare the organism for a secondary infection. The concept of ‘trained immunity’ suggests that, in addition to T and B cells, myeloid cells also acquire immunological memory through epigenetic changes in the chromatin [43]. According to this theory, danger signals in the form of PAMPs lead via PRRs to histone modifications not only at the promoter of target genes needed to combat invading pathogens, but also at enhancer sites. The initial interaction of the myeloid cell with the pathogen is thought to trigger chromatin remodeling events, leading to heightened H3K4me3 and H3K27ac marks at the respective gene loci, along with H3K4me1 marks at the corresponding enhancer sites. After elimination of the pathogen and termination of the immune response, the permissive histone marks on respective promoters are abolished, but the H3K4me1 persists as a latent enhancer. A myeloid cell of this type is trained to quickly induce gene expression for rapid defense responses and persists in a ‘resting state’ until it encounters a recurring pathogen [44]. Upon restimulation of the myeloid cell the promoter and the latent enhancer in the form of H3K27ac are modified, leading to increased gene expression via the effect of the activated latent enhancer.

### Consequences of viral infection on the host epigenome

Throughout history, viruses like SARS-CoV-2 have emerged, spreading globally and triggering pandemics that have claimed millions of lives. Challenged by severe disease courses, pandemics have detrimental effects on local health systems and long-term social as well as economic effects [45]. Viruses can hijack epigenetic mechanisms to control processes such as their life cycle and replication in host cells. It is not yet fully understood how exactly viruses induce these epigenetic changes, but it is clear that viruses intricately alter the functioning of host cells. Viruses influence epigenetic signals to favor viral gene production, thereby inhibiting the host gene expression in homeostatic conditions [46]. . Consequently, the host’s immune defenses are weakened, making the cells more susceptible to viral infection and disrupt signaling pathways of the host cell. Apart from these general aspects, the overall consequences of viral infections for aspects of epigenetic memory and trained immunity are not yet sufficiently understood. Further, epigenetic alterations can affect viral infections by two distinct means: (i) The host’s epigenetic changes can impact the host’s immunoregulation and affect the way the immune system responds to the virus, and (ii) epigenetic changes within the virus’ basic replication mechanism itself can alter the host immune response. To fully understand the underlying epigenetic mechanisms, the tropism of the virus and its replication process need to be investigated. This review summarizes the findings of chosen scientific studies that address these aspects.

Over the past 20 years, Next Generation Sequencing (NGS) techniques have improved constantly. Screening of open chromatin regions and identification of positions with permissive or repressive histone marks via ATAC-seq and multiplexing ChIP-seq improved the understanding of changes in chromatin modifications [47, 48]. Additionally, improved data analysis strategies are used to integrate diverse information on transcriptional and epigenetic levels also on single cell level [49–52]. Throughout the recent SARS-CoV-2 pandemic, immunologists collected data on epigenetic remodeling of immune cells during antiviral immune responses. In the following chapters, we summarize several of the most outstanding findings. Yet we are only beginning to understand the impact and consequences of epigenetic mechanisms in critical courses of coronavirus disease 2019 (COVID-19) courses or potentially in Long-COVID. Additionally, we will shed light on the latest knowledge on epigenetic influences during HIV infection and its consequences for the immune response and therapeutic approaches.

## Human immunodeficiency virus

In the early 1980s, HIV was identified and accepted as the causative agent of Acquired Immunodeficiency Syndrome (AIDS). However, it still presents a global threat to public health today [53, 54]. According to the World Health Organization, more than one million people acquired HIV in 2022 with over 600,000 HIV-related deaths [55]. HIV is a member of the *Retroviridae* family with two types: HIV-1 and HIV-2. Both types cause persistent infection with impairment of a regulated immune response and potential progression into AIDS. However, HIV-1 has been described as more virulent and the major cause of AIDS worldwide [56]. HIV-1 is primarily transmitted via unsafe heterosexual practices, with the sub-Saharan region of Africa accounting for the highest rates of HIV-1 infection [54].

### Characteristics of HIV infection

Structurally, the viral genome consists of two identical single-stranded RNA molecules enclosed by a capsid and the outer viral envelope. The viral envelope is formed by the glycoproteins gp120 and gp41 that bind to host cell surface receptors and therefore mediate the initial step in cell entry. Besides the RNA genome, the capsid carries viral enzymes that are essential for the HIV life cycle: The reverse transcriptase and integrase allow the conversion of viral RNA into DNA that can be permanently integrated into the host chromosome, and the protease cleaves protein precursors for the assembly of mature viral particles [56]. HIV-1 strains bind almost exclusively to the host cell surface receptor CD4 and one of two co-receptors, namely CXC Motif Chemokine Receptor 4 (CXCR4) or C-C Motif Chemokine Receptor 5 (CCR5), expressed on T-lymphocytes, monocytes/macrophages and DCs [56]. Following successful transmission, the virus is taken up by DCs at the site of infection and carried to the regional lymph node where CD4^+^ T cells are infected [57]. From there, the virus ultimately spreads into the bloodstream [56]. Infected monocytes circulate in the bloodstream and spread the virus into the gastrointestinal tract or the central nervous system (CNS), where perivascular macrophages and microglia account for the main HIV-1 reservoir [57].

Most HIV-related research has focused on the fate of HIV-infected (HIV+) CD4^+^ T cells. HIV infection results in drastic CD4^+^ T cell depletion, either caused by direct cell lysis following productive infection or by CD8^+^ T cell-mediated killing of infected cells [58], [58, 59]. Even non-permissive CD4^+^ T cells undergo cell death when unsuccessful viral replication results in the accumulation of cytosolic viral DNA transcripts triggering pyroptosis [60]. CD4^+^ T cell depletion and dysregulation of T cell homeostasis during disease progression have been suggested as the underlying mechanism of immunodeficiency [61]. In comparison to the CD4^+^ T cell reservoir, infected macrophages are less efficiently eliminated by CD8^+^ T cell-mediated killing [59]. Furthermore, HIV-1 infection in macrophages was shown to impair apoptotic host cell response [62].

Clinically, acute HIV infection manifests in heterogeneous flu-like symptoms that occur days to a few weeks after infection and are accompanied by high viremia levels and a significant decline in CD4^+^ T cell numbers. This is followed by a clinical asymptomatic period that can last for years. Here, the host mounts an immune response partly controlling viral replication and reducing HIV viremia. However, HIV replication is continuously ongoing leading to a state of chronic systemic inflammation and destruction of lymphoid tissue. Failure to restore a normal immune cell function under HIV infection encourages opportunistic infections and neoplastic diseases during the AIDS phase [56]. HIV + individuals are subjected to lifelong treatment with a combination of antiretroviral drugs that inhibit essential steps in the productive viral replication cycle. Combined antiretroviral therapy (cART) has been proven to successfully suppress viral replication, increase CD4^+^ T cell counts, and reverse clinically significant immunodeficiency, thus reducing morbidity and mortality. However, a major burden to complete eradication of the virus is latently infected cells that contain the integrated HIV genome and therefore form a persistent viral reservoir in infected patients [54]. Resting memory CD4^+^ T cells have already been established as a major latent viral reservoir [57]. Other researchers have pointed out that macrophages can also harbor the integrated HIV genome: However, it has been debated whether this cell population is an important long-lived HIV cell reservoir since monocyte-derived macrophages (MDMs) are usually short-lived and not capable of self-renewal. Conversely, tissue-resident macrophages derived from yolk-sac progenitors are agreed to be long-lived with self-renewable potential. As of now, it has not been thoroughly investigated which macrophage populations are chronically infected by HIV [63]. In HIV + patients under cART, integrated HIV-1 DNA could be isolated from urethral macrophages, intestinal macrophages and microglia [64–66]. These findings provide evidence that macrophages are a persistent HIV reservoir even under treatment. Taken together with their ability to withstand the cytopathic effects of HIV infection and form resting reservoirs in different tissues, myeloid cells might play a key factor in disease progression and the onset of comorbidities during HIV.

### Epigenetic regulation of myeloid cell functions in HIV infection

**Fig. 2:**
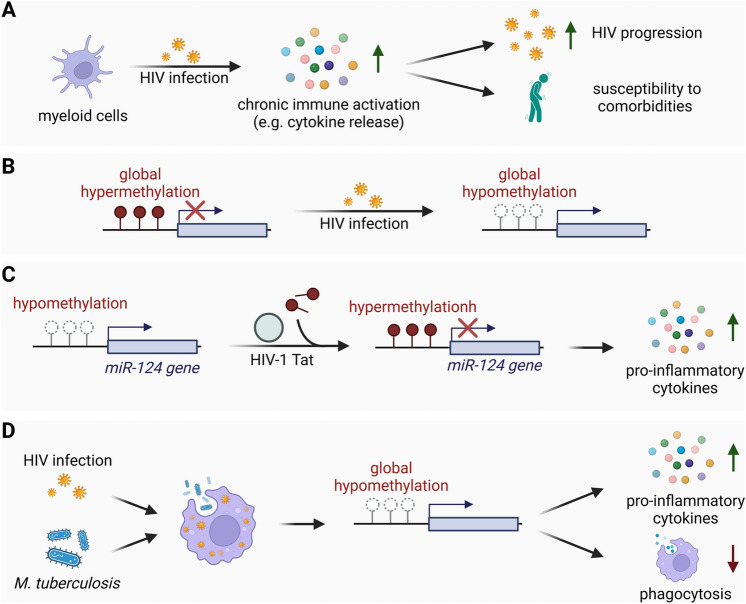
Epigenetic changes introduced into the myeloid cell host genome following HIV infection. **A** HIV infection of myeloid cells induces a chronic inflammatory state as seen in an excessive cytokine production that contributes to increased HIV progression and the development of comorbidities in infected individuals. **B** Acute HIV infection introduces a differentially hypomethylated DNA signature in isolated monocytes, including genes involved in innate immunity that can only be minimally restored after the onset of cART. **C** Stimulation with the HIV-1 Tat protein results in the hypermethylation of the miR-124 promoter and downregulation of miR-124 expression in primary microglia cells that correlates with microglial activation and increased release of pro-inflammatory cytokines. **D** Isolated monocytes from HIV + individuals challenged with M. tuberculosis present a hypomethylated DNA profile that is associated with increased pro-inflammatory cytokine production and impaired pathogen phagocytosis

One of the hallmarks of HIV infection is an aberrant systemic immune activation. This can be observed as a characteristic cytokine storm during acute infection, but the immune activation also persists throughout chronic infection despite effective antiretroviral treatment. Myeloid cells are believed to contribute to this abnormal immune activation by releasing excessive amounts of pro-inflammatory cytokines. This chronic immune activation is considered to drive HIV pathogenesis, as observed in the loss of CD4^+^ T cells and AIDS progression in infected individuals [67]. Additionally, chronic inflammation is assumed to be the major driving force behind the premature development of chronic non-infectious illnesses such as cardiometabolic diseases and cognitive dysfunction in HIV + individuals. These illnesses occur despite effective treatment and are usually associated with older age (Fig. 2A) [68].

Multiple research findings are hinting towards an association between a disrupted epigenetic profile and an activated inflammatory state in HIV + myeloid cell populations. Monocytes isolated from HIV + individuals were shown to exhibit differentially methylated loci that occur as early as acute HIV-1 infection [69]. Specifically, the affected gene loci were hypomethylated in HIV + individuals compared to uninfected controls indicating a transcriptionally active genomic state. Hypomethylated loci were found in genes critical for innate immune activation, including genes involved in the IFN response, such as the central inflammation-associated TFs Interferon Regulatory Factor 7 (*IRF7*) and Interferon Alpha Inducible Protein 27 (*IFI27*), suggesting an active inflammatory state in monocytes from HIV + patients. Importantly, the early onset of effective cART in infected patients was found to only marginally reverse the hypomethylated profile. This observation implies that HIV infection rapidly induces durable epigenetic changes potentially resulting in dysfunctional monocytes in the defense against infection (Fig. 2B) [69].

As stated before, chronic immune activation in HIV + individuals facilitates the onset of severe comorbidities even under effective treatment. One comorbidity associated with HIV infection is the development of insulin resistance. Insulin resistance is a major predisposition for the development of type 2 diabetes mellitus and a risk factor for developing cardiovascular diseases [70]. In monocytes, a comparison of the DNA methylation profile has shown that HIV + individuals characterized as insulin-resistant present hypomethylated DNA in comparison to insulin-sensitive individuals [71]. This hypomethylated DNA profile was associated with increased levels of pro-inflammatory cytokines like IL-1β, IFN-γ and Tumor Necrosis Factor (TNF)-α in HIV + insulin-resistant individuals. Monocytes are therefore suggested to present a differential epigenetic and inflammatory signature facilitating insulin resistance in HIV + individuals. Whether these different epigenetic signatures are fully associated with HIV infection status or dependent on other clinical risk factors remains to be elucidated.

In the CNS, ongoing neuroinflammation has been described as the underlying cause of HIV-1-associated neurological disorders (HAND) that can be observed in up to 60% of HIV + individuals under cART. Abnormal microglial activation upon HIV-1 infection is suggested to be a major driver of neuroinflammation [72]. The microglia quiescent state is essentially mediated by the miR-124. Consequently, downregulation of miR-124 expression is associated with microglial activation and neuroinflammatory pathogenesis. Recent studies have provided evidence that the HIV-1 Transactivator of Transcription (Tat) protein, an early viral protein involved in viral transcription, induces the downregulation of miR-124 in primary microglia cells via DNA methylation. HIV-1 Tat stimulation was shown to induce the expression of DNA methylation enzymes and increase DNA methylation of the miR-124 promoter in microglial cells, resulting in reduced expression of miR-124 [73]. This HIV-1 Tat-induced hypermethylation of the miR-124 promoter and following downregulation of miR-124 expression was later shown to correlate with the exaggerated release of proinflammatory cytokines from primary microglia (Fig. 2C) [74].

Adding to research on HIV-related neuropathogenesis, Corley and colleagues investigated the epigenetic profile in monocytes of HIV + individuals experiencing cognitive impairment (CI) [75]. CI describes restrictions in behavioral, cognitive, and motor functions that can be observed in around 40% of HIV + individuals and is thought to be mediated by neuroinflammation and neuronal apoptosis. HIV + monocytes from the periphery are believed to indirectly contribute to neuroinflammation since they can migrate into the CNS and mediate virus spread to resident microglia and astrocytes. Monocytes from HIV + individuals with CI were found to exhibit differentially methylated loci (DML) compared to non-CI controls [75]. Specifically, the majority of the DML was shown to be hypermethylated in monocytes from HIV + individuals with CI. Genes that were differentially methylated included the brain-derived neurotrophic factor (*BDNF*) and fibroblast growth factor 2 (*FGF2*) genes that are hypothesized to be associated with neurocognitive impairment. Furthermore, various genes involved in the inflammatory response, including the *IL-7* gene, were differentially methylated in monocytes from HIV + individuals with CI. The exact molecular mechanisms of how a dysregulated epigenetic profile in peripheral monocytes in HIV + individuals contributes to the progression of neuropathogenesis remains to be determined [75].

Another risk factor for HIV + individuals is their increased vulnerability to secondary infections with bacterial and viral pathogens such as *Mycobacterium tuberculosis* (*M. tuberculosis*) and Hepatitis C Virus (HCV). Despite the observed immune activation, HIV + patients fail to efficiently eliminate the co-pathogens which is why those secondary infections can often have a serious and even fatal course of disease [76, 77]. Alveolar macrophages serve as an essential immune defense against respiratory pathogens in the lung and have recently become subject to HIV-related research since HIV + patients display a greater risk of developing pulmonary diseases. A pilot study from Staitieh and colleagues identified differences in the accessibility of certain gene loci and concordant gene expression in alveolar macrophages of HIV + patients compared to uninfected individuals [78]. In this study, over 100 genes were identified that exhibited higher accessibility of several chromatin regions in alveolar macrophages of HIV + patients. This increased chromatin accessibility has been shown to correlate with elevated gene expression, including genes involved in pro-inflammatory immune responses mediated by cytokines such as C-X-C Motif Chemokine Ligand 2 (*CXCL2*), *CXCL3* and *IL-1β*. Although this study was limited to a low number of participants, these findings suggest an association between epigenetic alterations and an activated inflammatory state in alveolar macrophages in HIV + individuals. The authors hypothesize that a chronic immune activation might lead to an exhausted state of alveolar macrophages that could facilitate chronic lung diseases observed in patients with HIV [78]. This assumption is especially interesting since Espindola and colleagues have proposed that an exhausted inflammatory state of monocytes isolated from HIV + individuals might impair their capacity to efficiently clear *M. tuberculosis* infection [79]. In vitro experiments revealed a significantly exaggerated production of pro-inflammatory cytokines in monocytes isolated from HIV + patients when challenged with *M. tuberculosis* in comparison to uninfected controls. Interestingly, monocytes isolated from HIV + patients simultaneously showed impaired phagocytosis and a profound deficit in *M. tuberculosis* killing compared to uninfected controls. *M. tuberculosis*-challenged monocytes from HIV + individuals were shown to have reduced global DNA methylation levels, again suggesting a link between a differentially methylated epigenetic signature and a dysregulated immune response. Interestingly, *M. tuberculosis* challenged monocytes from HIV + patients with severe disease progression presented significant DNA hypomethylation, upregulation of activating chromatin remodeler such as the histone acetyltransferase 1 (HAT1) and increased production of pro-inflammatory cytokines including IL-6 and IL-1β compared to HIV + patients with low disease progression. It is argued that with ongoing disease progression, the aberrant immune activation of monocytes results in an exhausted state that impairs a directed and efficient immune response towards co-pathogens (Fig. 2D) [79].

**Fig. 3:**
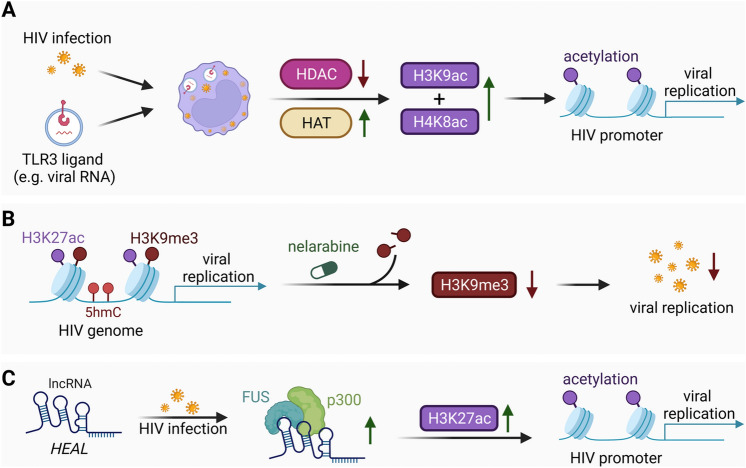
Epigenetic regulation of the integrated HIV genome in infected myeloid cells. **A** In vitro stimulation of a monocytic U38 cell line, which carries the stably integrated HIV-1 promoter, with TLR3 ligands showed an overall decreased HDAC activity and increased HAT activity. As a result, H3K9ac and H4K8ac marks at the HIV promoter were increased, which was shown to correlate with increased promoter activity. **B** The integrated HIV genome in MDMs presents a unique epigenetic signature composed of the bivalent histone signature H3K9me3, H3K27ac and 5hmC methylation throughout the HIV genome that mediates continuous viral expression. Treatment with a novel purine analogue named nelarabine results in reduced H3K9me3 levels and suppressed viral replication in HIV + macrophages. **C** HIV-1 infections lead to in vitro upregulation of the host lncRNA *HEAL* in primary MDMs. Subsequently, *HEAL* forms a complex with the RNA-binding protein FUS and recruits the HAT p300 to the HIV-1 promoter, resulting in increased H3K27ac levels and HIV expression

Further, HIV + individuals are prone to simultaneous infection with HCV due to similar disease transmission. Bhargavan and colleagues suggested that the co-infection with RNA viruses such as HCV might contribute to HIV replication and pathogenesis via epigenetic alterations in myeloid cells of HIV + individuals: In vitro stimulation of latent HIV + monocytic cell lines with ligands of the Toll-like receptor 3 (TLR3), a sensor of foreign nucleic acid structures, was shown to rapidly induce acetylation of the integrated HIV promoter [80]. Specifically, H3K9ac and H4K8ac were introduced which were associated with increased HIV-1 promoter activity. Consistent with these findings, TLR3 stimulation also resulted in a global decrease in HDAC activity and an increase in HAT activity (Fig. 3A) [80].

### Epigenetic modulation of the integrated HIV genome

In addition to the gene loci of the host, Lu and colleagues found a unique epigenetic signature of the HIV genome in human in vitro differentiated MDM distinct to a latently infected T cell line that is especially observed in the genome of positions of the integrated HIV genome [81]. Compared to latently infected T cells, the integrated viral genome in MDMs was shown to be highly enriched with H3K9me3, a mark usually associated with condensed chromatin, despite active transcription of the viral genome. This H3K9me3 mark was found to occur in combination with H3K27ac across the HIV genome. H3K27ac is usually associated with transcriptionally active chromatin and together with H3K9me3 forms an unusual bivalent histone signature in HIV + MDMs. Additionally, MDMs presented an enrichment of 5’-hydroxymethylcytosines (5hmCs), which are associated with gene activation, throughout the HIV genome compared to the viral genome in latently infected T cell reservoirs [81]. While the exact epigenetic regulation and interplay of different histone modifications throughout the HIV genome in myeloid cells remains to be fully understood, these findings support a differential regulation of the HIV genome between the myeloid and T cell reservoir (Fig. 3B).

Building on these results, Yi and colleagues presented a novel correlation between disruption of epigenetic regulation and successful suppression of HIV-1 expression in macrophages [82]. Treatment of HIV + macrophages with a purine analog named nelarabine was demonstrated to successfully suppress viral gene expression, which correlated with reduced H3K9me3 levels on the viral genome as well as cellular genomic loci [82]. This suggests that the identified unique epigenetic signature of the HIV genome in myeloid cells might lead to novel therapeutic approaches to disrupt viral expression (Fig. 3B).

Adding to this, Chao and colleagues identified a novel lncRNA named HIV-1 enhanced lncRNA (*HEAL*) in HIV + MDMs that is believed to promote persistent HIV-1 replication via epigenetic regulation. *HEAL* was shown to be upregulated in both MDMs and microglia by HIV-1 infection, which correlates with increased HIV-1 replication. *HEAL* is proposed to form a complex with the RNA-binding protein FUS and bind directly to the HIV-1 promoter, effectively recruiting the HAT p300 from the host’s transcription factor pool, which results in increased H3K27ac of the HIV-1 promoter and HIV expression. Targeting *HEAL* with therapeutic molecules might support suppression of viral expression (Fig. 3C) [83]. On the contrary, the binding of FUS to G4s located in the LTR region of HIV-1 has been found to downregulate viral transcription [84].

Alamer and colleagues determined a small molecule modulator named ZL0580 as a potentially novel therapeutic agent to epigenetically suppress HIV expression in various myeloid cell populations [85]. ZL0580 modulates the chromatin-reader protein bromodomain-containing protein 4 (BRD4) resulting in two different mechanisms of HIV suppression in myeloid cells. BRD4 induced by ZL0580 was shown to inhibit productive recruitment of the viral Tat protein to the HIV promoter region in microglial as well as monocytic cell lines. Additionally, treatment of microglia cells with ZL0580 resulted in a more repressive chromatin structure at the LTR of HIV, which was associated with suppressed HIV expression in microglia [85].

So far, the current therapeutic strategy using cART has proven to have limited efficacy for the persistent HIV reservoir in myeloid cells. Understanding the unique epigenetic regulation of viral expression in myeloid cells is a promising tool for identifying potential therapeutic molecules that successfully suppress HIV expression.

## Severe acute respiratory syndrome coronavirus type 2

SARS-CoV-2 belongs to the *Coronaviridae* family and is one of several coronaviruses (CoVs) that are infectious to humans. CoVs can be categorized into four genera: α‑CoVs, β‑CoVs, γ‑CoVs, and δ‑CoV. All are capable of infecting mammals; only γ-CoVs and δ-CoV can also infect avian species. CoVs are enveloped viruses containing positive-sense single-stranded RNA (ssRNA) and nucleocapsids with helical symmetry [86]. SARS-CoV‑2 is classified as a novel β‑CoV, which emerged in Wuhan (People’s Republic of China) in December 2019. It spread rapidly and globally, causing the COVID-19 pandemic with over 772 million officially registered cases and more than 6.9 million deaths worldwide as of November 19, 2023 [87, 88]. This respiratory illness primarily affects cells of the pulmonary system, which can lead to pneumonia and multi-organ failure while triggering a series of immune responses that can control and eliminate the virus [89]. While most infected individuals have mild to moderate symptoms, some infected patients (approximately 1%) have a critical, sometimes fatal, progression [87]. Common symptoms of this infection are fever, headache, loss of taste and smell, and other severe complications [86]. The incubation period of the virus is estimated to be about 5 to 12 days, and transmission occurs through aerosol infection of the respiratory tract, particularly when coughing and sneezing, but also through close contact with infected individuals [89].

### Characteristics of SARS-CoV-2 infection

Like other RNA viruses, the SARS-CoV-2 genome is susceptible to random mutations over time that can affect both its structural and non-structural genes. As a result, various SARS-CoV-2 variants have evolved since 2019, some of which have propagated efficiently. Five of these variants have been classified by the World Health Organization as variants of concern (VOC) during the pandemic, with Alpha (B.1.1.7), Beta (B.1.351), Gamma (P.1) and Delta (B.1.617.2) now classified as de-escalated variants, and Omicron (line B.1.1.529) as one of the VOCs still circulating [89]. During a severe COVID-19 course, the body’s immune system quickly mounts the production of various pro-inflammatory cytokines, such as IL‑1β, IL‑6 and TNF‑α in the blood. This phenomenon termed ‘Cytokine Storm’ can lead to intense inflammation, damage to the lungs, acute respiratory distress syndrome (ARDS), and failure of multiple organs [90]. As of January 2023, Nirmatrelvir/Ritonavir (a combination of protease inhibitors) is strongly recommended for non-severe COVID-19 courses. On the contrary, corticosteroids (e.g. Dexamethasone), which are used to decrease the severity of a cytokine storm, e.g. in sepsis, IL-6 receptor inhibitors (e.g. Tocilizumab or Sarilumab) and Baricitinib, a Janus kinase (JAK) inhibitor used to inhibit the intracellular signaling of cytokines, (individually or in combination) are recommended for severe to critical disease severity [91].

Central to the pathogenesis of SARS-CoV-2 is its interaction with the Angiotensin-Converting Enzyme 2 Receptor (ACE2R) facilitating its entry into human cells. This involves the Spike (S) protein of SARS-CoV-2 binding to the ACE2R, while the host cell Transmembrane Serine Protease 2 (TMPRSS2) and the Paired Basic Amino Acid Cleaving Enzyme Furin (FURIN), a proteolytic enzyme, cleave the S protein into its S1 and S2 subunits, facilitating the fusion of viral and cellular membranes and thereby permitting the virus to release its RNA into the cell [92].

### Epigenetic properties of host cells in SARS-CoV-2 infection

**Fig. 4:**
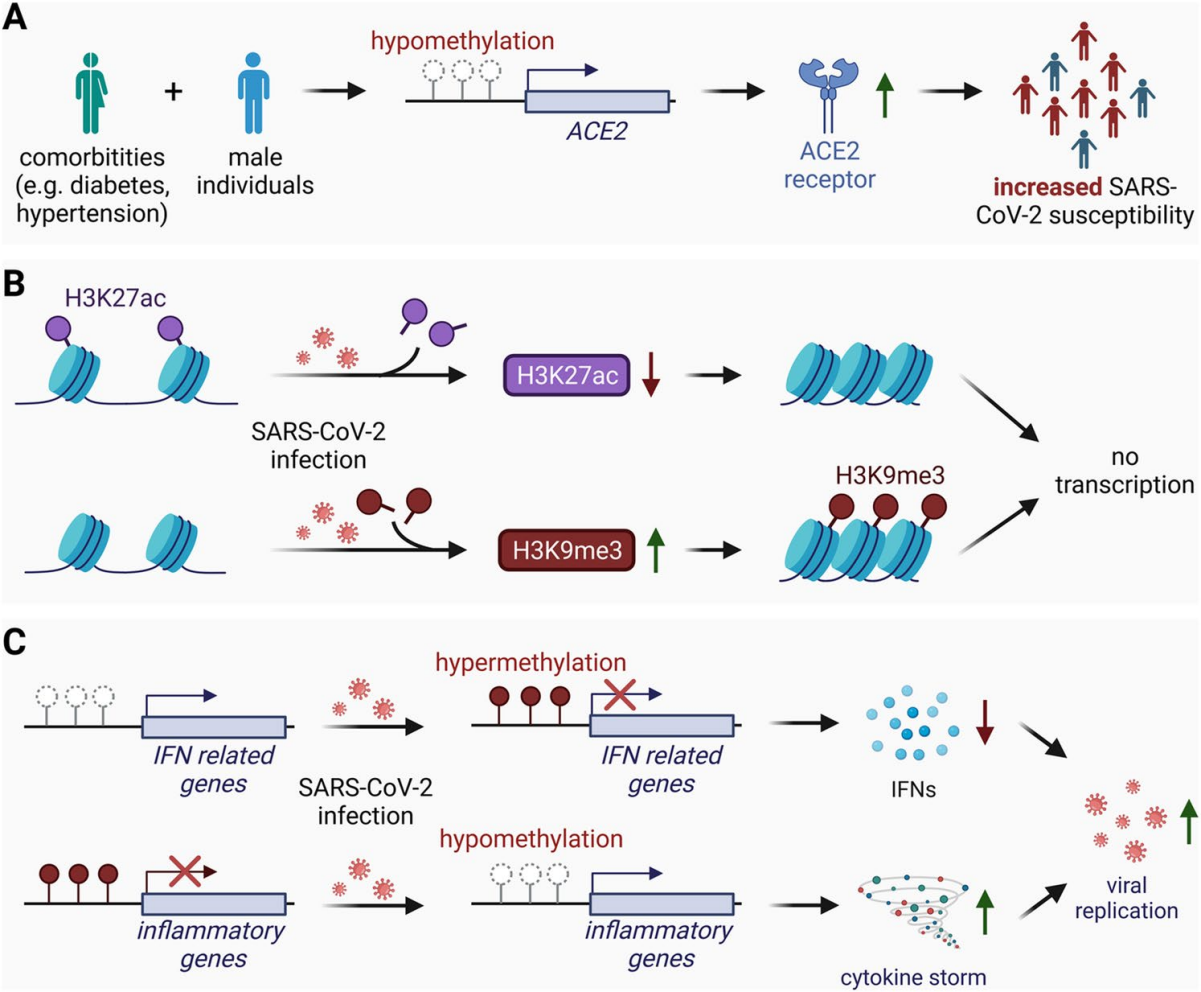
Epigenetic signatures in SARS-CoV-2 pathogenesis. **A** Variations in the methylation patterns of the *ACE2* gene in different cells show a correlation between hypomethylation and increased ACE2R expression in males and individuals with comorbidities in contrast to females and healthy individuals. This suggests a molecular basis for the observed increase of SARS-CoV-2 susceptibility. **B** After SARS-CoV-2 infection of human lung adenocarcinoma A549 cells, a consistent decrease of the active histone mark H3K27ac and an increase of the repressive histone mark H3K9me3 can be observed, indicating direct infection-related epigenetic changes. **C** Genome-wide DNA methylation profiles of peripheral blood mononuclear cells from critically COVID-19 patients revealed hypermethylation in regions associated with the IFN response and hypomethylation in regions associated with inflammation and cytokine production

The Angiotensin-Converting Enzyme 2 gene (*ACE2*), which encodes the receptor ACE2R, has thus become a hotspot of epigenetic research. Preliminary studies by Corley and Ndhlovu have revealed variations in the methylation patterns of the *ACE2* gene in different cells and found an interesting correlation between age and sex-specific hypomethylation tendencies [93]. In general, DNA methylation of *ACE2* was found to be lowest in lung cells compared with other cell types, suggesting increased ACE2 expression in these cell types. Other studies have confirmed that ACE2Rs are differentially expressed in various human cell types. They are predominantly found in lung alveolar epithelial cells and the small intestine [94]. This finding is consistent with the fact that the lung is the primary organ affected, and in severe cases, pneumonia, ARDS, and other serious respiratory complications occur. Additionally, the finding of hypomethylation in males, but not females, provides a potential molecular basis for the observed increased susceptibility and severity of disease and mortality in men (Fig. 4A) [95, 96]. Another study identified a correlation between highly increased ACE2 expression and heightened severity of COVID-19 with comorbidities such as hypertension, diabetes, cerebrovascular disease, coronary heart disease, pulmonary disease, and smoking (Fig. 4A) [97].

Diving deeper into the regulatory architecture of ACE2, it is evident that the gene’s expression is not just a simple on-off switch. Instead, it is modulated by a complex network of epigenetic controls including but not limited to DNA methylation [98]. Pinto and colleagues uncovered several epigenetic regulators in human lung tissue, described below, that contribute to the histone modifications associated with *ACE2* expression [97]. On the one hand, they identified modifiers such as HAT1 and HDAC2 as responsible for regulating histone acetylation of *ACE2* gene regions. HDAC2 is an important regulator of the well-known H3K27ac mark, an active histone mark for accessible chromatin regions that is associated with increased gene expression. On the other hand, several genes with a positive correlation to ACE2 were found to be regulated by Lysine Demethylase 5B (KDM5B), a histone demethylase, highlighting the role of histone methylation in COVID-19 pathogenesis. KDM5B modulates histone methylation specifically of lysine 4 at histone 3 (H3K4), which includes the prominent active histone marks H3K4me1, H3K4me3, and the H3K27ac mark enabling gene transcription [97]. Taken together, existing epigenetic variations in host *ACE2* expression inevitably influence SARS-CoV-2 virulence. In addition, Wang and colleagues also observed a consistent and significant decrease in the active histone mark H3K27ac and an increase of heterochromatin mark H3K9me3 following SARS-CoV-2 infection of human lung adenocarcinoma cells A549, indicating epigenetic modification by infection (Fig. 4B) [99].

Some researchers have found that DNA methylation patterns in regions associated with IFN responses are hypermethylated and regions associated with inflammation and cytokine production are hypomethylated in peripheral blood mononuclear cells of severely ill SARS-CoV-2 infected patients (Fig. 4C) [100]. These findings match the observed phenotype of severely diseased individuals: downregulated antiviral IFN response, hyperactive inflammation processes and increased cytokine secretion - a cytokine storm [101]. In line with this, Castro de Moura and colleagues identified more than 40 CpG sites associated with the severity of SARS-CoV-2 infection, with most of these genes primarily involved in the IFN response [102]. Moreover, Godoy-Tena and colleagues found CpG sites specifically associated with IFN genes and antigen presentation in peripheral blood monocytes from severely ill SARS-CoV-2 infected patients [103]. These results demonstrate an epigenetic and transcriptional reprogramming of monocytes in peripheral blood that may be associated with the release of abnormal immature monocytes, elevated systemic amounts of pro-inflammatory cytokines, and alterations in immune cell communication in these individuals. The identification of such DNA methylation sites could also serve as potential biomarkers as well as prove valuable in  the clinical stratification and treatment of individuals infected with SARS-CoV-2.

Moreover, DNA methylation also appears to play a role in post-SARS-CoV-2 infection. It is known that an increase in methylation events is often associated with enhanced immune responses to the defense of the host. However, the presence of a distinct post-infection methylation pattern (e.g. after a mild SARS-CoV-2 infection) has been proposed to increase an individual’s susceptibility to the virus, which is paradoxically detrimental for the host. It is likely that these post-infection methylation patterns predict outcomes, possibly due to decreased viral suppression from down-regulated IFN activity [104]. In another independent study, the abundance of hyper- and hypomethylated sites in monocytes from convalescent COVID-19 patients was screened using epigenome-wide association studies (EWAS) [105]. The researchers observed that some minor transcriptomic alterations in monocytes persist together with the methylation profile in convalescent SARS-CoV-2 patients. Overall, this suggests that persistent SARS-CoV-2 methylation may indicate a residual epigenetic scar in myeloid cells.

Further, the inactivation of the X chromosome in mammals (X-chromosome inactivation; XCI) is a prime example of the interplay of epigenetic mechanisms, specifically DNA methylation, histone modifications and non-coding RNAs, regulating gene expression and creating stable cellular states. XCI is essential for maintaining the balance of gene dosage between men and women. It describes the process in which one of the two X chromosomes in female mammals becomes transcriptionally silenced, a complex and strictly regulated event occurring during early embryonic development [106]. This process of XCI also participates in COVID-19 pathogenesis and several other diseases. During this process, however, genes can “escape” inactivation, meaning that certain genes have a higher expression level in women than in men with only one active X chromosome. XCI is suspected to participate in COVID-19 pathogenesis by altering the expression of the TLR7 gene, which is located on the X chromosome [107]. The endosomal TLR7 receptor is pivotal for the recognition of viral single-stranded RNA and part of the viral sensing machinery of the innate immune response inducing the production of type I IFNs. In addition, increased expression of TLR7 leads to increased production of type I IFNs. The *TLR7* gene has been found to escape the epigenetic silencing, which can lead to sex-specific diseases. Given this, Asano and colleagues determined X-linked recessive TLR7 deficiency as a risk factor for COVID-19 pneumonia in men [108]. It is known that this gene dosage effect can be caused by the escape of XCI [109]. Such epigenetic variations between sexes not only highlight the complexity of our genomic responses to pathogens but also underscore the potential for personalized medical approaches in the future. As we further investigate the relationship between the X chromosome and disease susceptibility, a promising pathway for tailored therapeutic strategies emerges. Further research is crucial, as understanding these differences could greatly improve possibilities for novel therapeutic approaches for COVID-19.

Another epigenetic mechanism involved in the immune response to SARS-CoV-2 is the regulation by miRNAs, which belong to the group of ncRNAs. The interactions of ncRNA with mRNA result in the formation of complexes with specific proteins that lead to the degradation of mRNA and the subsequent inhibition of protein synthesis. Additionally, ncRNAs facilitate the recruitment of proteins that modulate histones and thereby influence the transcriptional status of genes by activating or repressing their expression. In general, miRNAs can have contrasting influences on host immunity as epigenetic regulators: They can either play a role in combating the viral threat (antiviral) or function as proviral factors. It is hypothesized that mRNAs can not only induce the host’s immune response but also benefit viral pathogenesis by assisting in viral immune evasion [110]. For instance, antiviral properties of miRNAs are suppressing the entry of the virus, preventing the spread of virions, and minimizing the systemic symptoms caused by the infection. In this context, Khan and colleagues predict some host miRNAs with antiviral properties to play a role in SARS-CoV‑2 infection, such as has-miR-17‑5p and has-miR-20b‑5p [111]. Additionally, circulating miRNAs, like has-miR-155, has-miR‑208a and has-miR‑499, were found to be increased in critically ill SARS-CoV-2 infected individuals [112]. Has-mirR-208a and has-miR-499 were previously found to be associated with myocardial/cardiomyocyte damage and have been observed in up to 57% of hospitalized COVID-19 patients [113]. Has-miR-155 has been shown to be a key regulator of innate and adaptive immune response, targeting genes associated with defense against viral infections such as Influenza A virus and SARS-CoV-2 [114, 115]. Although COVID-19 and Influenza-ARDS patients, for example, have similar symptoms, the two groups can interestingly be distinguished by differences in the concentrations of has-miR-155 and has-miR-499 [112]. This observation identifies both miRNAs as unique epigenetic regulators of a SARS-CoV-2 infection. Understanding these mechanisms may help to unravel new therapeutic interventions, such as designing RNA interferences (RNAi) to silence post-transcriptional gene modifications or miRNA mimics to increase/restore the function of specific miRNAs.

### Modulation of the epigenetic machinery of the host by SARS-CoV-2

In addition to the influence of host epigenetic properties on the replication and spreading of the SARS-CoV-2 virus, there are epigenetic alterations mediated by the virus itself. SARS-CoV‑2 has been shown to generate viral miRNAs that could epigenetically influence host cells as a proviral mechanism. However, the exact epigenetic mechanistic roles of these miRNAs in the pathogenesis of COVID-19 are not yet fully established [116]. Using computational analysis of miRNA-mediated interactions in SARS-CoV-2 infection, several possible viral miRNAs and their potential effects were highlighted. An important observation was that the virus primarily targets the transcription of host genes associated to autophagy, IFN-I signaling, and mTOR signaling [111]. In addition, it is assumed that viral miRNAs could target transcripts for TFs associated with important cellular processes, such as transcription, metabolism and various crucial signaling pathways. For example, Signal Transducer and Activator of Transcription 1 (*STAT1*), *STAT5B* and SRY-Box Transcription Factor 11 (*SOX11*) have been identified as target genes for predicted viral miRNAs. Consequently, inhibition of STAT family members by miRNAs could potentially lead to an impairment of the IFN response and a shift towards enhanced viral replication via miRNAs. This knowledge could lead to the development of anti-miRNAs, so-called antagomirs, targeting viral miRNAs to suppress endogenous miRNA activity [116].

**Fig. 5:**
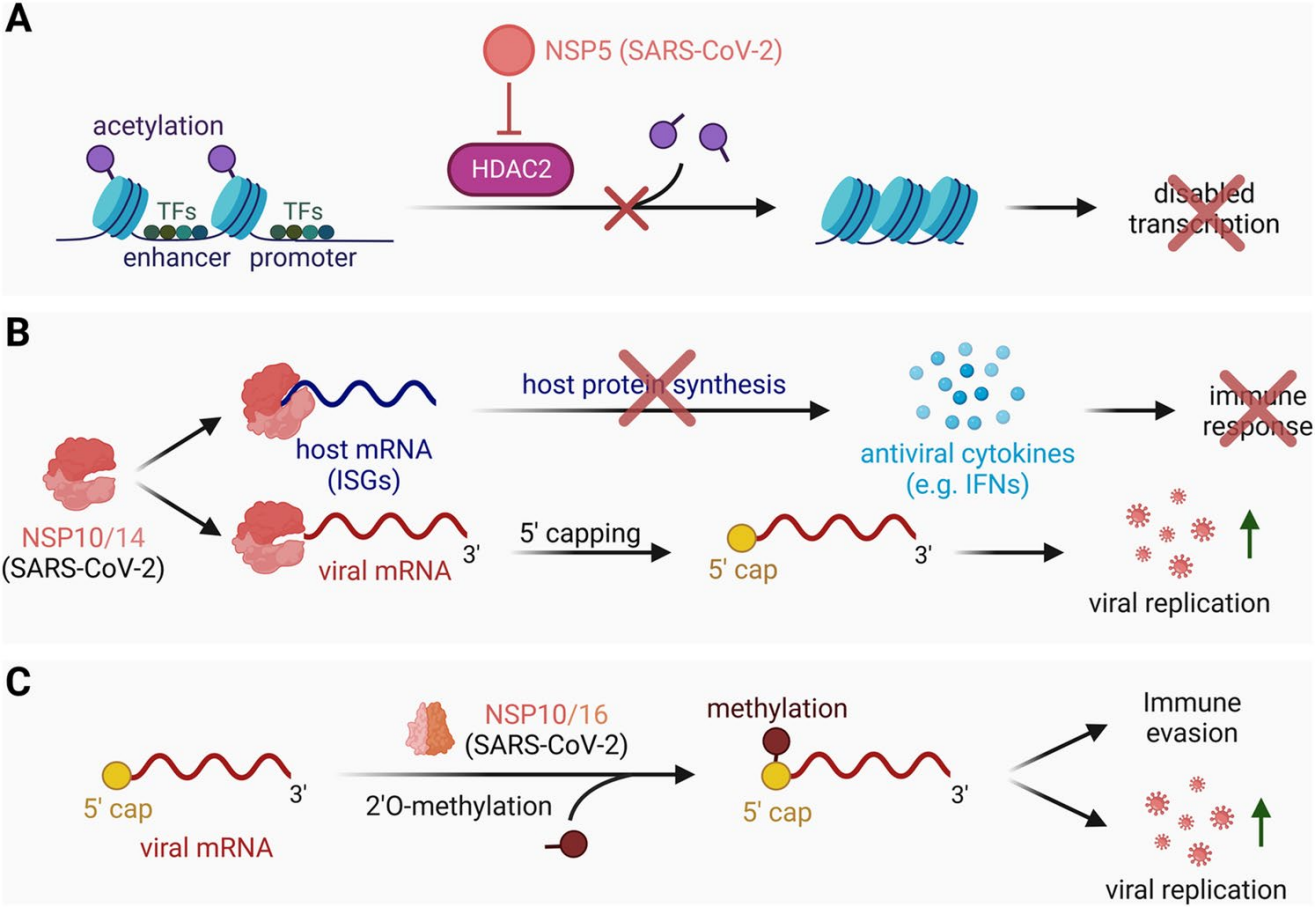
Viral strategies targeting host epigenetic machinery and translation regulation in SARS-CoV-2. **A** The NSP5 of SARS-CoV-2 interferes with the epigenetic regulation of the host cell by interacting with HDAC2, leading to inhibition of acetylation degradation and subsequent dysregulated transcription. **B** NSP14, in complex with NSP10, suppresses host mRNA translation leading to the downregulation of ISGs and consequently inhibited IFN release, and caps the 5′ end of viral RNA to protect viral mRNA and ensure viral replication. **C** The NSP10 and NSP16 complex adds a methyl group to the viral RNA Cap-1 structure with its 2′-O-methyltransferase activity, helping to camouflage viral RNA from the innate immune system, ensuring RNA stability, and promoting efficient protein production

Intensive research on the replication mechanisms of SARS-CoV-2 has revealed additional epigenetic regulatory mechanisms of the virus that alter the host epigenome: In its effort to replicate and dominate, the virus can trigger profound epigenetic alterations in the host. Multiple non-structural proteins (NSPs) seem to play a role in altering the epigenome. SARS-CoV-2 encodes 16 NSPs (NSP1-16), of which NSP5, NSP10, NSP14 and NSP16 have been investigated thoroughly to date. NSP5 was shown to potentially affect the transport of proteins into the endoplasmic reticulum and mitochondria by interacting with the host epigenetic regulator HDAC2 [117]. In more detail, researchers predict an interaction of NSP5 with the cleavage site at the nuclear localization of HDAC2 [117, 118]. Upon binding of NSP5, the transport of HDAC2 into the nucleus is inhibited, resulting in a lack of chromatin closure (Fig. 5A). Hence, the epigenetic regulation via HDAC2 would be affected.

NSP14, an exoribonuclease and N7-methyltransferase (N7-MTase) was found to be a novel inhibitor of host translation [119]. Exoribonucleases degrade RNA by progressively removing nucleotides from its ends, contributing to cellular RNA quality control and turnover. N7-MTases are enzymes catalyzing the transfer of a methyl group to the N7 position of guanine in RNA and hence play a crucial role in RNA molecule modification. Studies suggest that the complex of NSP14 together with NSP10 has a triple function: (i) It ensures proper viral RNA translation through its proofreading activity, (ii) it simultaneously represses the translation of host mRNAs, and (iii) it caps the 5’ end of viral RNA via its N7-MTase activity (Fig. 5B) [119–121]. In addition, NSP10/NSP14 have been found to inhibit protein expression of a broad spectrum of Interferon-stimulated genes (ISGs), such as retinoic acid-inducible gene I (RIG-I), Tripartite motif-containing protein 21 (TRIM21) and Radical S-adenosyl methionine domain-containing protein 2 (Viperin), through its global translation inhibition ability [119]. This activity confers additional protection to the virus against the IFN response. While the exact process of the translation inhibition is not fully understood yet, the study by Hsu and colleagues indicates that the ability of NSP14 to interfere with host protein production is likely key to the virus’ role in immune evasion.

Other NSPs help to camouflage viral RNA from recognition by the innate immune system: The NSP10/NSP16 complex functions as a 2’-O-methyltransferase complex that adds a methyl group to the viral RNA Cap-1 structure. The 2′-O-methylation in the Cap-1 structure of viral RNA is believed to help evade the innate immune response, ensure RNA stability, and enable efficient protein production (Fig. 5C) [122, 123]. Hence, NSP10, as a cofactor for multiple SARS-CoV-2 enzymes, like NSP14 and NSP16, could be a promising future target for therapeutic opportunities, i.e., for the development of molecules that can affect its structure or disrupt its interaction with other NSPs.

A non-classical epigenetic mark is the N6-methyladenosine (m^6^A) modification of RNA, which is an important gene regulatory mechanism and is also known as RNA methylation. Liu and colleagues reported that m^6^A is abundant in SARS-CoV-2 RNA as well as in host RNA. Host mRNA methylation appears to be directly affected by SARS-CoV-2 infection: The m^6^A methylome is dynamically changed post-infection [124]. The researchers found an increase in m^6^A in both the viral genome and host mRNA. Furthermore, the effects of m^6^A modifications seem to be rather negative and hinder SARS-CoV-2 spread. However, it is unclear if the viral or host m^6^A modifications are responsible for suppressing the viral replication [124]. Manipulating this modification could be a strategy to combat the virus by developing an attenuated vaccine strain or developing new antiviral ‘epidrugs’ that target m^6^A.

Another fascinating concept in the field of epigenetics in the context of SARS-CoV-2 infection is histone mimicry. This concept refers to the phenomenon of non-histone proteins adopting modifications typically associated with histones. These modifications can include methylation, acetylation, ubiquitination, and others. Mimicking these histone-like post-translational modifications (PTMs) in non-histone proteins can significantly affect cellular processes. The work of Kee and colleagues highlights a novel case of histone mimicry during SARS-CoV‑2 infection [125]. Specifically, expression of the SARS-CoV-2 protein Open Reading Frame 8 (ORF8) was observed to interfere with histone PTM regulation by mimicking regions of human histone protein H3 containing post-translational modifications required for transcriptional regulation. Hence, ORF8 enhances chromatin compaction resulting in increased viral replication [125]. In essence, this histone mimicry can perturb the cell’s normal regulatory mechanisms, potentially conferring a strategic advantage to the virus within the host cellular environment.

## Conclusion

Over the last 20 years, our understanding of epigenetic modifications in myeloid cells and their consequences for myeloid function has improved dramatically. New concepts such as trained immunity are challenging the field to overcome dogmas and improve our understanding of myeloid cell plasticity.

The presented conjoined research findings provide evidence that HIV infection of diverse myeloid cell populations results in broad epigenetic alterations. These alterations promote a chronically activated state that potentially drives disease progression and changes effective response to common co-infections. In this context, it has been pointed out that the aberrant inflammatory state of myeloid cells might be a driving cause of HIV-related comorbidities and non-AIDS mortalities. Myeloid cells further present a unique epigenetic regulation of viral expression that differs from the latent T cell reservoir and provides new promising therapeutic targets for the suppression of viral expression. Further investigations of the epigenetic regulations of host and viral genomes in myeloid cells is needed to elucidate their contribution to HIV progression and the onset of severe comorbidities.

In combating SARS-CoV-2, a holistic understanding of both the virologic intricacies and the nuanced epigenetic mechanisms is crucial. The interplay between the virus and the host cellular machinery, influenced by epigenetic regulations, offers insights into potential viral vulnerabilities and the host’s adaptive capabilities. Despite the improved knowledge of these interactions, the complexity of the conditions will lead to further and more intensive investigations of the role of myeloid cells in SARS-CoV-2 pathogenesis. The deeper we explore these interactions, the more opportunities for novel therapeutic interventions like ‘epidrugs’ will become apparent. Interdisciplinary research efforts combining virology and epigenetics could be the basis for a reliable response to this global health challenge.

Current findings on the pathology of SARS-CoV-2 and HIV reveals the significant role of host epigenetics in viral infections, alongside the impact of viral epigenetic mechanisms on the host immune system. The identification of potential therapeutic targets, coupled with intervention strategies involving ‘epidrugs’ to modify the epigenetic marks, holds promise for treating persistent viral reservoirs and associated complications. This knowledge deepens our comprehension of viral pathogenesis and guides the development of tailored therapeutic strategies in the ongoing battle against infectious disease.

## Data Availability

No datasets were generated or analysed during the current study.

## Abbreviations

5hmC 5′: hydroxymethylcytosine
5mC 5: methylcytosine
ac: acetylation
ACE2: Angiotensin-Converting Enzyme 2
AIDS: Acquired Immunodeficiency Syndrome
ARDS: Acute respiratory distress syndrome
C5: 5th carbon position of cytosine
cART: combined antiretroviral therapy
CI: Cognitive impairment
CNS: Central nervous system
CoV: Coronavirus
DNMT: DNA methyltransferase
H3: Histone 3
H4: Histone 4
HAT: Histone Acetyltransferase
HCV: Hepatitis C Virus
HDAC: Histone deacetylases
HIV: Human Immunodeficiency Virus
HIV+: HIV-infected
IFN: Interferon
IL: Interleukin
ISGs: Interferon-stimulated genes
K: Lysine
lncRNA: Long non-coding RNA
LTR 5′: 5′ long terminal repeat
m6A: N6-methyladenosine
MDM: Monocyte-derived macrophages
me: methylation
me3: tri-methylation
miRNA/miR: microRNA
mRNA: messenger RNA
ncRNA: non-coding RNA
NSP: Non-structural protein
PAMPs: Pathogen-associated molecular patterns
PRRs: Pattern recognition receptors
PTMs: Post-translational modifications
SARS-CoV-2: Severe Acute Respiratory Syndrome Coronavirus
scRNA: single-cell RNA
seq: sequencing
SNP: Single nucleotide polymorphism
ssRNA: single-stranded RNA
Tat: Transactivator of transcription
TF: Transcription factor
TLR: Toll-like receptor
TNF: Tumor Necrosis Factor
UTRs 3′: 3′-untranslated regions
VOC: Variant of concern
XCI: X-chromosome inactivation
COVID-19: Coronavirus disease 2019
CpG 5′: Cytosine-phosphate-Guanine-3′
DAMPs: Damage-associated molecular patterns
DCs: dendritic cells
